# On Intermissions and Irregularities of the Pulse, as Caused by the Imperfections of the Mitral Valves

**Published:** 1829-04-01

**Authors:** 


### On Intermissions and Irregularities of the Pulse, as caused by Imperfections of the Mitral Valves. By Dr. Hodgkin.

A discussion on the above subject, in the Westminster Medical Society, during the session preceding the present one, must be in the memory of most of its members. While Dr. Johnson was urging his opinion, that intermissions and irregularities of the pulse were mainly dependent on disturbed function or structure of the mitral valves, the worthy sub-editor of the Lancet, who was then permitted to mingle in medical society, got up, and stated (on his honour and veracity, of course) that the experience of Dr. Hodgkin led him to an opposite conclusion—namely, that the intermissions and irregularities above-mentioned were connected with disease of the aortic, that is to say, with the semilunar valves. The following quotation from Dr. Hodgkin's paper, published in the Medical Gazette, will add another proof to the many which we have put on record, how completely the fountain of truth and science has been contaminated by the Wakleys and Lamberts of our days—who, we verily believe, never did, and never will, utter a single syllable of truth—except by mere accident, and when, of course, they are unaware of such a transaction.*

"Before I conclude, I shall take the liberty of adding, as a small semeiological contribution, a few remarks on the indication of valvular disease, and therefore not altogether foreign to the subject before us.

"Of all the valvular diseases, the affections of the mitral are attended by the most characteristic symptoms. *When these valves are sufficiently deranged to produce a disturbance of function, there is great irregularity of the pulse*, which, if not always present, is at least observable whenever the circulation is disturbed by exercise or any other excitement. In affections of the aortic valves there is generally *no irregularity or intermission of the pulse ;* nevertheless intermissions are not very unfrequent.

"The comparison of the pulse with the action of the heart itself is very essential, and tends to throw the clearest light on these affections. In derangements of the mitral valve, the beat of the heart cannot be better characterized than by the term tumultuous. It baffles all attempts to analyse its rythm.

"In affections of the aortic valves, on the contrary, the action of the heart is generally, if not always, regular; and even when intermissions are perceived at the wrist, the lost pulsations may be felt at the heart. In diseases of the aortic valves, auscultation often detects a prolonged and perverted sound, such as has been compared to the stroke of a saw, the puff of a pair of bellows, or the action of a rasp.

"This distinction between the affections of the mitral and aortic valves I have found invariably confirmed by numerous cases. Such, indeed, might have been judged to be the case *à priori*. When the mitral valve is so diseased as to disturb its function, there are two orifices by which the ventricle may discharge its contents; and it must depend on the almost fortuitous varieties of the action of the valve, as well as on the degree of fulness of the auricle, which must be influenced by every cause capable of affecting the circulation, whether the contraction of the ventricle should propel the blood by its usual course through the semilunar valves into the aorta, or by a retrograde movement through the mitral valve into the auricle. The circumstances are very different in the case of disease of the aortic valves, the mitral remaining sound. Here the circulation is disturbed by no reflux from the ventricle, and every contraction must propel the blood through the aorta, although the force of the contraction may not be sufficiently great to render the impulse perceptible in the remote branches. These considerations, confirmed by experience, induce me to differ from Corvisart and some other authors who have treated of the diseases of the heart, and who regard irregularity of the pulse as peculiarly characteristic of disease of the aortic valves. I have never

* In a recent sitting of the London Medical Society, Dr. Ramadge got up, and declared that the import of what he had said was *erroneously* reported in the Lancet of the preceding week. Mr. Wakley makes Dr. Ramadge say, and even repeats it, that the "report in the Lancet was *perfectly correct*." Such is the editor, and such the sub-editor of the Lancet! Men whose trade or occupation it is, to annihilate or conceal truth, and to earn their bread by inventing and recording falsehoods. Such is the surgical science of the Lancet! The editor is aware that no medical man believes one word he says—and, therefore, his dependence is on the non-professional public, who are incapable of detecting either the ignorance or mendacity of statements in the said publication.

found myself deceived by the symptoms which I have described as indicative of disease of the mitral valves; and I am hence induced to place the derangements of this part amongst those internal affections which may be pronounced upon with the least uncertainty.

"I am thine very truly,

"Thomas Hodgkin."

With the foregoing observations we agree entirely, and we are happy to find that Dr. Hodgkin has put another proof on record of the mendacity of Lambert.

